# Geriatric assessment in the older adult with genitourinary cancer: A narrative review

**DOI:** 10.3389/fonc.2023.1124309

**Published:** 2023-02-02

**Authors:** Surbhi Singhal, Julianna G. Marwell, Ali Raza Khaki

**Affiliations:** ^1^ Division of Medical Oncology, Department of Medicine, Stanford University, Stanford, CA, United States; ^2^ Section of Geriatric Medicine, Division of Primary Care and Population Health, Department of Medicine, Stanford University, Stanford, CA, United States

**Keywords:** geriatric assessment, older adult, bladder cancer, kidney cancer, prostate cancer

## Abstract

Genitourinary (GU) cancers including bladder, prostate, and kidney cancers affect older adults with a higher prevalence compared to younger adults. GU cancer treatment is associated with poorer outcomes in older adults compared to their younger counterparts. To better identify and support older adults receiving cancer care, oncologic societies recommend the use of a geriatric assessment (GA) to guide management. However, little is known about the implementation and usefulness of the GA in older adults with GU cancers. We performed a narrative review to investigate the utility of the GA in older adults with GU cancers and propose strategies to optimize the real-world use of the GA. Here, we describe a framework to incorporate GA into the routine cancer care of older adults with GU cancers and provide several implications for future research.

## 1 Introduction

Many genitourinary (GU) cancers occur primarily in older adults with a median age at diagnosis of 72 years for bladder cancer (1), 66 years for prostate cancer (1), and 64 years for kidney cancer (1). Older adults with GU cancers undergoing cancer therapy have overall poorer outcomes including greater impairment in health-related quality of life (2), higher rates of chemotherapy toxicity (3), and lower cancer-specific and overall survival (4–6) than their younger counterparts. However, older adults are a heterogeneous population and these outcomes in older adults may be driven by their pretreatment functional status and comorbidities, instead of chronological age alone (7, 8). Given the growing need to better identify and support older adults receiving cancer care, the American Society of Clinical Oncology (ASCO), the National Comprehensive Care Network (NCCN), and the International Society of Geriatric Oncology (SIOG) recommend the use of a geriatric assessment (GA) to guide management of older adults (9–11). In this review, we first summarize the relevant literature regarding the use of the GA in older adults with cancer and then review relevant literature for GA in GU cancers and propose settings in which the GA may best support clinical decision-making.

## 2 Methods

We conducted a literature search of PubMed for articles published in English up to November 21, 2022. There were three major components of the keyword and subject heading search linked with the AND operator: GU cancer terms, including bladder, prostate, and kidney cancer; older adult terms, including frailty and elder; geriatric assessment terms, including instrument, tool, geriatric domains, and implementation. We reviewed the titles and abstracts to identify research articles that described the implementation of geriatric assessment in older adults with GU cancers. We also conducted a separate manual literature review, including conference proceedings, to ensure appropriate capture of relevant studies.

## 3 Geriatric assessment for adults with cancer

**Figure 1:**
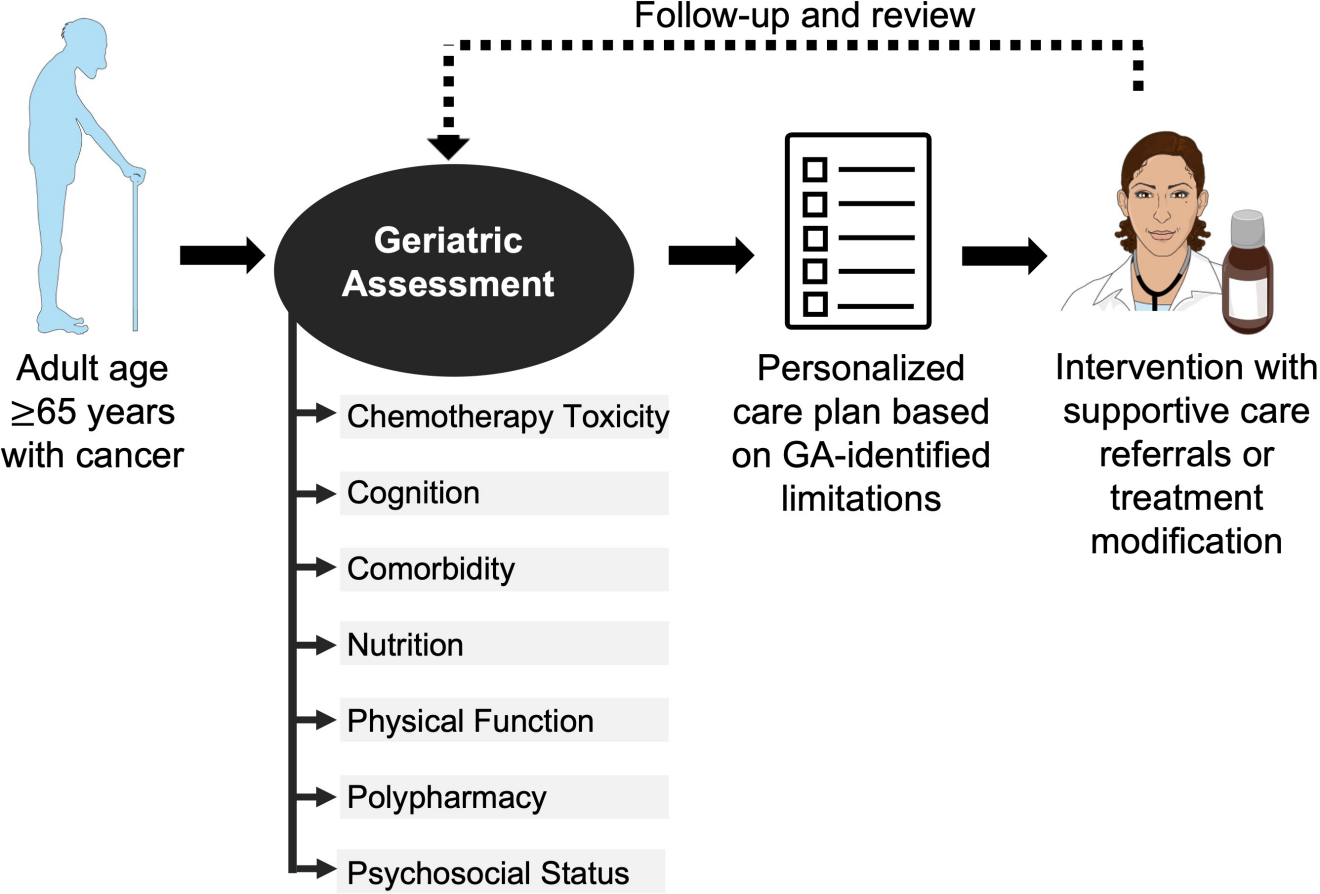
Use of geriatric assessment in management of older adult with cancer. The American Society of Clinical Oncology recommends all adults with cancer ≥65 years starting chemotherapy undergo a geriatric assessment of multiple domains to guide management. The Figure was partly generated using Servier Medical Art, provided by Servier, licensed under a Creative Commons Attribution 3.0 unported license.

The GA is a multidimensional holistic assessment of older adults’ medical, psychosocial, and physical functioning (12). Some form of GA can be used as a tool to assess patient fitness for surgery or radiation and to guide chemotherapy management. The gold standard is for a trained team member to perform a GA of multiple geriatric domains before chemotherapy initiation for adults age ≥ 65 years (**Figure 1**) (11). The results of this GA can inform a personalized care plan, which could include a myriad of interventions such as supportive care referrals, pre-habilitation for surgery, patient education, or treatment modification (9). The (**Table 1**) summarizes exemplar common instruments used to evaluate the GA domains in older adults with cancer. Geriatric domains commonly evaluated include risk for chemotherapy toxicity, cognition, comorbidity, nutrition, physical function, polypharmacy, and psychosocial status.

**Table 1:** Exemplar geriatric assessment instruments in genitourinary (GU) cancer.

Instrument	Content	Scoring	Use in GU Cancers
Chemotherapy Toxicity			
Cancer and Aging Research Group Chemotherapy Toxicity Tool (CARG-TT) (3)	Clinician-assessed and patient-reported 11 items: sex, age, cancer type, chemotherapy dosage, number of chemotherapy agents, creatinine, hearing, falls, falls in past 6 months, assistance with medications, walking ability, and social activity	Scored from 0-23 points, where higher score confers greater risk. Risk for Grade 3 or higher chemotherapy toxicity: 0-5: 30% (low risk) 6-9: 52% (intermediate) 10-23: 83% (high)	Patients with prostate cancer undergoing chemotherapy or ADT (13)
Chemotherapy Risk Assessment for High-Age Patients (CRASH) (14)	Hematologic risk: chemotherapy risk, diastolic blood pressure, LDH, and IADLs Non-hematologic risk: MNA, ECOG performance status, and mini mental health status	Scored from 0-12 points, where higher score confers greater risk. Risk for Grade 4 hematologic toxicity or Grade 3-4 non-hematologic toxicity: 0-3: Low risk 4-6: Intermediate-low risk 7-9: Intermediate-high risk ≥10: High risk	
Galsky Criteria (15)	ECOG performance status ≥2, creatinine clearance <60 mL/min, ≥grade 2 hearing loss, ≥grade 2 neuropathy, and/or NYHA Class III heart failure	Presence of any feature makes patient unfit for cisplatin-based chemotherapy	Patients with advanced urothelial carcinoma undergoing consideration of cisplatin
Gupta Criteria (16)	ECOG performance status ≥3, creatinine clearance <30 ml/min, ≥grade 2 peripheral neuropathy, NYHA Class III heart failure	Presence of any feature makes patient unfit for platinum-based chemotherapy	Patients with advanced urothelial carcinoma undergoing consideration of platinum chemotherapy
Cognition			
Mini-Cog (17)	Clinician-assessed 3 items: 3-word registration, clock drawing, and 3-word recall	Scored from 0-5 points, where lower score confers higher risk. Patients with score of <3 points should undergo dementia screening.	Patients with prostate cancer undergoing consideration of chemotherapy or ADT (18, 19) Patients with kidney cancer undergoing consideration for TKIs (20) Strongly correlates with MMSE, but faster to complete (21)
Mini Mental State Examination (MMSE) (22)	Clinician-assessed 11 items: 5 items on language and praxis, 2 items on orientation, and 1 item each on registration, attention and calculation, recall, and copying	Scored from 0-30 points, where lower score confers higher risk. ≥25: normal cognition <24: impaired cognition	Patients with prostate cancer undergoing consideration of chemotherapy or ADT (18, 19) Patients with kidney cancer undergoing consideration for TKIs (20)
Comorbidity			
Adult Comorbidity Evaluation 27 (ACE-27) (23)	Clinician-assessed 26 items of presence and severity of individual medical illnesses	Scored from 0-3 points, where higher score confers greater severity in comorbidity. Degree of comorbidity: 0: none 1: mild 2: moderate 3: severe	Patients with MIBC undergoing consideration of RC (24)
Charlson Comorbidity Index (25)	Clinician-assessed 17 items of presence of individual medical illnesses	Scored from 0-37 points, where higher score confers higher risk for 10-year mortality.	Patients with nonmetastatic prostate cancer undergoing consideration of RP (26, 27) Patient with nonmetastatic kidney cancer undergoing consideration of PN or RN (28)
Cumulative Illness Rating Scale-Geriatric (CIRS-G) (29)	Clinician-assessed 14 items of presence of individual medical illnesses	Scored from 0-56 points, where higher score confers higher severity.	Patients with prostate cancer undergoing consideration of chemotherapy (18) Patients with kidney cancer undergoing consideration for TKIs (20)
OARS Comorbidity Questionnaire (30)	Patient-reported 26-items of presence of 13 individual medical illnesses and the degree to which they impair patient’s activities	Scored from 0-39 points, where higher score confers higher comorbidity burden.	
Health Status Screening			
Geriatric 8 Questionnaire (G8) (31)	7-item screening tool: food intake, weight loss, body mass index, mobility, neuropsychological problems, prescription medications, and self-perception of health	≤14: Proceed with full GA >14: Full GA not required	Patients with prostate cancer undergoing consideration of new therapy (32)
Nutrition			
Malnutrition Universal Screening Tool (MUST) (33)	Clinician-assessed 3 items: BMI, weight loss, and presence of acute disease	Scored from 0-6 points, where higher score confers higher risk. Management guidelines: 0: Routine clinical care 1: Observe ≥2: Treat with referral to dietitian	Patients with MIBC undergoing consideration of RC (34) Use as correlative factor for sarcopenia assessment (35)
Patient-Generated Subjective Global Assessment (PG-SGA) (36)	Patient-reported assessment across 4 domains: weight, food intake, symptoms, activities and function	Scored from 0-36 points, where higher score confers higher risk. Management guidelines: 0-1: No intervention required. 2-3: Education with pharmacologic intervention as indicated by symptom survey 4-8: Dietitian intervention ≥9: Critical need for improved symptom management and/or nutrient intervention	
Physical Function			
OARS Instrumental Activities of Daily Living (IADL) (30)	Patient-reported assessment in dependence across 7 items: using the telephone, shopping, navigating transportation, preparing meals, doing housework, and managing medicine and money	Scored from 0-14 points, where lower scores confer higher IADL dependence	Patients with prostate cancer undergoing consideration of chemotherapy (18) Patients with kidney cancer undergoing consideration for TKIs (20)
Polypharmacy			
Medications self-report (37)	Patient-reported number of medications including prescriptions, over the counter medications, and herbal supplements	Higher number of medications confer higher risk for poylpharmacy. Polypharmacy most frequently defined as ≥5 regularly scheduled medications.	Patients with kidney cancer undergoing consideration for TKIs (20)
Psychosocial Status			
Geriatric Depression Scale (GDS) (38)	Patient-reported 15 items	Scored from 0-15 points, where higher scores confer higher risk for depression. Score ≥5 suggests depression.	Patients with prostate cancer undergoing consideration of chemotherapy (18) Patients with kidney cancer undergoing consideration for TKIs (20)
Lubben Social Network Scale (LSNS) (39)	Patient-reported 12 items assessing social engagement, including family and friends	Scored from 0-60 points, where lower scores confer less social engagement	Patients with prostate cancer undergoing consideration of chemotherapy (18)

BMI, body mass index; ECOG, Eastern Cooperative Oncology Group; GA, geriatric assessment; GU, genitourinary; IADL, instrumental activities of daily living; LDH, lactate dehydrogenase; MIBC, muscle invasive bladder cancer; MNA, mini nutritional assessment; NYHA, New York Heart Association; OARS, Older Americans Resources and Services; PN, partial nephrectomy; RC, radical cystectomy; RN, radical nephrectomy; RP, radical prostatectomy; TKI, tyrosine kinase inhibitor.

The use of the GA to guide management of older adults with cancer has been shown to increase likelihood of cancer treatment completion (40), reduce hospitalizations and emergency room visits (when paired with geriatrician co-management) (41), and improve patient-centered communication and enhance satisfaction for older adults and their caregivers (42). The pivotal clinical trial, GAP70+, demonstrated lower treatment toxicity with GA. This randomized clinical trial enrolled 718 adults ≥70 years of age with incurable solid tumors or lymphoma (15% with GU cancers) across 40 community oncology practices (43). The practices were randomized 1:1 to the intervention group where oncologists received a tailored GA summary with specific recommendations including patient education, medication review, supportive care referrals, and/or chemotherapy modification or the usual care group where no GA summary or recommendations were provided to oncologists. Fewer patients in the intervention group experienced grade 3-5 toxic effects compared with the usual care group (51% versus 71%, p<0.001), with no effect on overall survival between the groups at 6 and 12 months (43).

## 4 Bladder cancer

### 4.1 Bladder cancer in the older adult

At least 75% of bladder cancer cases are diagnosed in adults age ≥65 years (1). Age is not only a risk factor for development of bladder cancer, but also is a risk factor for increased mortality with bladder cancer (44).

The treatment approach for non-muscle invasive bladder cancers (NMIBC) is based on tumor risk stratification, with standard of care curative management ranging from single instillation of intravesical chemotherapy for low-risk tumors to 1-3 years of bacillus Calmette-Guerin intravesical instillation or consideration of radical cystectomy (RC) for high-risk tumors (45). For patients with nonmetastatic muscle invasive bladder cancer (MIBC), the standard of care curative treatment is RC preceded by neoadjuvant cisplatin-based chemotherapy for those who are cisplatin-eligible (45). However, RC is associated with high morbidity and mortality, with a 90-day complication rate up to 59% and 90-day mortality rate up to 5% (46). Organ-preservation using definitive radiation with chemotherapy is an alternative option for curative management generally reserved for patients who prefer an alternative to RC or those considered unfit for surgery (45, 47).

Metastatic bladder cancer is treated with non-curative intent with sequential systemic therapies with the goal to help patients extend and/or improve their quality of life. Standard of care first-line therapy includes platinum-based chemotherapy regimens with decision stratification based on cisplatin eligibility (45). Subsequent therapy options include immune checkpoint inhibitors targeting programmed cell death 1 (PD-1) or its ligand (PD-L1) given either as switch-maintenance therapy for those without progression of disease on platinum-based chemotherapy or as a salvage agent at the time of progression; followed by antibody-drug conjugates targeting nectin-4 (enfortumab vedotin) and trop 2 (sacituzumab govitecan). For patients with FGFR2 or FGFR3 alterations, erdafitinib, an oral tyrosine kinase inhibitor (TKI) can also be utilized as a salvage post-platinum therapy (45, 48).

### 4.2 Geriatric assessment in bladder cancer

Limited studies describe the implementation or usefulness of a GA in older adults with bladder cancer. One group piloted implementation of a GA in their Bladder Cancer Multidisciplinary Clinic with 94 patients and found high GA component completion rates (79-100%) with low rates of perceived burden by patients (49). The authors plan to evaluate associations between individual GA instruments, treatment decisions, clinical outcomes, and quality of life as their future work. A separate group implemented a GA in older adults ≥65 years of age with distal urinary tract cancer (25%) and prostate cancer (75%) undergoing perioperative management and found that patients with localized bladder cancer had higher pretreatment comorbidities and greater frequency of postoperative complications than patients with prostate cancer (50).

Previous studies identify risk factors for adverse outcomes after bladder cancer treatment, which we apply to create recommendations regarding GA in older adults with bladder cancer (**Table 1**). Older adults who underwent RC for MIBC had higher mortality risk if they had severe comorbidity as assessed by the Adult Comorbidity Evaluation 27 (ACE-27) (23) or sarcopenia as assessed by radiologist review of axial computerized tomography images (24, 34). While sarcopenia assessment can be time and labor intensive, nutritional status assessment *via* the Malnutrition Universal Screening Tool (MUST) (33) or Patient-Generated Subjective Global Assessment (PG-SGA) (36) could serve as a correlative marker for sarcopenia when evaluating older adults for RC (35).

Cisplatin is a foundational chemotherapy in bladder cancer, with prior studies demonstrating improved disease response rates and overall survival with cisplatin compared to carboplatin when treating metastatic urothelial carcinoma (51, 52). However, cisplatin is associated with increased renal toxicity in older adults (53, 54). It is therefore recommended to perform an assessment of risk for chemotherapy toxicity prior to treatment initiation for older adults. While this can be done using the Cancer and Aging Research Group Chemotherapy Toxicity Tool (CARG-TT) (3) or Chemotherapy Risk Assessment for High-Age Patients (CRASH) (14) tools, the Galsky criteria was developed as a consensus definition of cisplatin eligibility for use in patients with metastatic urothelial carcinoma and has been applied in clinical trials and general practice to determine whether a patient is unfit for cisplatin-based chemotherapy (15). Recent efforts defined similar consensus criteria for platinum-based chemotherapy in the current treatment era (**Table 1**) (16).

## 5 Prostate cancer

### 5.1 Prostate cancer in the older adult

Similar to bladder cancer, prostate cancer is a disease with much higher prevalence in older adults. The median age at prostate cancer diagnosis is 66 years, with 70% of deaths occurring in adults ≥75 years of age (1).

Expected patient survival and prostate cancer risk stratification guide treatment decisions for nonmetastatic prostate cancer, which can include active surveillance, focal ablation, radical prostatectomy (RP), external beam radiation therapy, or brachytherapy, with or without androgen deprivation therapy (ADT) (55). Like RC for bladder cancer, (RP) for prostate cancer is a highly morbid procedure with 52% of patients reporting urinary incontinence and 59% of patients reporting sexuality problems two months after surgery (56).

Metastatic prostate cancer is treated with non-curative intent, with the foundation of treatment being ADT. Men treated with ADT can have multiple deleterious effects including higher risk for metabolic syndrome and diabetes (57, 58), coronary artery disease (59), fractures (60), and cognitive dysfunction (61). In addition to ADT, further systemic treatment options include oral agents targeting testosterone axis (e.g. abiraterone, enzalutamide, etc.), cytotoxic chemotherapy (e.g. docetaxel, cabazitaxel), radioactive isotopes (Radium-223) or radionuclide therapy (Lu 177 vipivotide tetraxetan), PARP inhibitors (e.g. olaparib or rucaparib for patients with BRCA1, BRCA2, or ATM alterations) or immunotherapy (e.g. pembrolizumab for patients with MSI-high or TMB-high tumors) (55). Taxane chemotherapy in older men with prostate cancer is associated with risk of infections, thromboembolic events, and hospitalization (62).

### 5.2 Geriatric assessment in prostate cancer

Several studies describe the use of a GA to guide management in older adults with prostate cancer. A multicenter study implemented a GA encompassing five geriatric domains prior to initiation of docetaxel in 24 patients ≥70 years of age with metastatic castrate resistant prostate cancer (18). The study found that frail patients, defined as those who had impairment in either Instrumental Activities of Daily Living (IADL) (30), Cumulative Illness Rating Scale-Geriatric (CIRS-G) (29), Geriatric Depression Cale (GDS) (38), Mini Mental State Examination (MMSE) (22), or Lubben Social Network Scale (LSNS) (39) identified by the GA, were more likely to discontinue docetaxel chemotherapy early compared to non-frail patients (60% versus 13% early discontinuation rate, p=0.037). Two other studies prospectively administered a GA to older adults planning to undergo radiation therapy for nonmetastatic prostate cancer, which did not identify older adults at risk for significant acute radiation toxicity (63) or subsequent diminished quality of life (64).

Another team used the Geriatric 8 questionnaire (G8) (31) to screen older adults with prostate cancer for subsequent GA but did not report its utility to predict outcomes in the study population (65). In 2017, SIOG published updated guidelines for the management of older adults with prostate cancer and recommend use of the G8 to categorize older adults into one of four categories prior to selecting a specific treatment: “healthy or fit” (G8 >14), “frail” (G8 ≤ 14, but conditions are reversible based on GA), “disabled or with severe comorbidities” (nonreversible conditions based on GA), or “terminally ill” (32). Patients in the “healthy or fit” or “frail” categories were considered fit for standard prostate cancer treatments and SIOG recommended selection of treatment be based on shared-decision making. On the other hand, the authors recommended adults in the “disabled or with severe comorbidities” category have specific geriatric interventions as guided by the GA to improve fitness but did not provide explicit details on the type or frequency of intervention. As a screening tool, the G8 could be useful to quickly identify high versus low-risk older adults.

Previous studies identify risk factors for adverse outcomes after prostate cancer treatment, which we apply to create recommendations regarding GA in older adults with prostate cancer (**Table 1**). Older adults who underwent RP for nonmetastatic prostate cancer had higher postoperative complications, late urinary complications, surgery-related death, and mortality if they had high pretreatment comorbidity, defined as Charlson Comorbidity Index of ≥2 (26, 27). Docetaxel is frequently used when cytotoxic chemotherapy is indicated for metastatic prostate cancer treatment. The CARG-TT can predict chemotherapy toxicity and has been externally validated to predict ≥grade 3 toxicity in men ≥65 years of age undergoing chemotherapy or ADT for metastatic castrate-resistant prostate cancer (13). We therefore recommend assessing comorbidity for patients undergoing consideration for RP and CARG-TT for patients undergoing consideration for chemotherapy or ADT.

## 6 Kidney cancer

### 6.1 Kidney cancer in the older adult

The median age at kidney cancer diagnosis is 64 years, with older adults with metastatic kidney cancer having worse overall survival than their younger counterparts (1, 66).

The standard of care treatment approach for nonmetastatic kidney cancer is either partial (PN) or radical nephrectomy (RN) with consideration of treatment with the immune checkpoint inhibitor pembrolizumab for patients with intermediate to high-risk disease, including those with stage ≥2 tumor with nuclear grade 4 or sarcomatoid differentiation, stage ≥3 tumor, regional lymph node metastasis, or stage M1 with no evidence of disease (67). Ablative techniques are reserved for very small tumors or for patients who prefer an alternative to nephrectomy or are unfit for surgery (68, 69). RN is associated with high rates of incident chronic kidney disease in up to 69% in one retrospective study (70). Metastatic kidney cancer is treated with non-curative intent systemic therapy with immune checkpoint inhibitors, TKIs, or a combination, with treatment selection guided by the International Metastatic Renal Cell Carcinoma Database Consortium prognostic model and patient-specific characteristics (68, 71). Subsequent treatment lines include additional TKIs or mTOR inhibitors. The immune checkpoint inhibitor combination of ipilimumab targeting cytotoxic T-lymphocyte-associated protein 4 (CTLA-4) and nivolumab targeting PD-1 is used in metastatic kidney cancer but has high rates of treatment-related toxicity with immune-related adverse events (72). On the other hand, the TKIs have high rates of bleeding and cardiovascular complications (73–75).

### 6.2 Geriatric assessment in kidney cancer

Studies describe the use of a GA in older adults with metastatic kidney cancer receiving TKIs. A retrospective single-institution study of 86 adults age ≥70 years treated with first line sunitinib or pazopanib for metastatic kidney cancer evaluated the ability of a pretreatment GA to predict subsequent treatment toxicity and outcomes (20). The authors used the GA to categorize patients into either a “fit”, “vulnerable” or “frail” category on the basis of five domains: IADLs, GDS, MMSE, CIRS-G, and polypharmacy. The pretreatment GA was able to differentiate patients at risk for grade 3 to 4 treatment-related toxicity, shorter progression free survival and shorter overall survival (20). However, a separate study using pretreatment GA in adults with a median age of 74 years starting sunitinib for metastatic kidney cancer found no correlation between pretreatment frailty as assessed by the GA and subsequent treatment toxicity or disease response (76). We did not identify any studies evaluating the role of GA to predict immunotherapy toxicity specifically for patients with metastatic kidney cancer. Further studies are needed in kidney cancer to explore the utility of the GA to determine future treatment toxicity.

Previous studies identify risk factors for adverse outcomes after kidney cancer treatment, which we apply to create recommendations regarding GA in older adults with kidney cancer (**Table 1**). Adults age ≥75 years with clinical T1 kidney tumors had worse overall survival if they had higher comorbidity based on the Charlson Comorbidity Index (28). A subsequent Medicare study of older adults with T1a kidney cancer who were nephrectomy candidates reported that compared to RN, treatment with PN was associated with greater overall survival, which was the most beneficial for patients with high pretreatment comorbidity (Charlson Comorbidity Index ≥1) on subgroup analysis (77). We therefore recommend comorbidity evaluation for patients with nonmetastatic kidney cancer who are considering either PN or RN.

## 7 Limitations of GA research in older adults with GU cancers

While it is encouraging to see some early studies showing implementation and utility of GA for patients with GU cancer, the studies identified have several limitations. Most studies were non-randomized, did not include control comparator arms, and had a limited sample size, which makes interpretation of patient quality of life and health outcomes with GA implementation challenging. Further, there remains limitations regarding generalizability due to substantial heterogeneity across several domains including GA instrument and delivery, patient demographics, and treatment type. Also, the GA was most frequently conducted at a single timepoint, which precluded capture of longitudinal geriatric outcomes during cancer treatment. After completion of the GA, few studies explicitly detailed the type of GA-informed supportive care intervention or treatment modification performed. This is likely because no evidence-based standard exists to guide interventions after GA, which could be an opportunity for future research. Although GAs have been successfully implemented and used to guide management of older adults with cancer, research of the GA in older adults with GU cancers remains preliminary and would benefit from further studies, including robust clinical trials.

## 8 Recommendations for GA use in the clinical setting

GAs have not been widely adopted into routine clinical practice with common barriers including lack of time, lack of training and knowledge, and the GA being too cumbersome (78, 79). In light of these challenges, pending prospective validation to verify feasibility and utility in the real-world setting, we propose the following recommendations:

- Use screening instruments, such as the 7-item G8 (31), to identify which older adults would most benefit from a GA.
- Evaluate comorbid conditions and nutritional status for older adults considering surgery.
- Assess risk for chemotherapy toxicity, physical function, polypharmacy, and psychosocial status for high-risk older adults considering chemotherapy.
- Screen for cognitive decline in older adults considering ADT or chemotherapy.
- The results of this focused GA can be applied to inform fitness for surgery and systemic therapy, guide referrals to supportive care services, and allow for patient-centered shared-decision making.

## 9 Conclusions

Geriatric assessment is a valuable clinical tool with the potential to better personalize care delivery for patients with cancer. Patients with GU cancers may be a particularly suitable population for GA implementation, given the high prevalence of older adults and the variable treatment considerations in both the localized and metastatic setting based on patient fitness and treatment tolerance. Through this narrative review, we describe the usefulness of the GA in older adults with GU cancers and propose strategies to optimize the real-world use of the GA. As we identify in this review, the literature regarding GA implementation often comes from studies including patients with multiple cancer types, which may not capture the nuance of treatment decisions in patients with specific cancers. Furthermore, there remains a paucity of evidence of the use of GA for specific treatment decisions outside of systemic chemotherapy. Ideally the GA tools would be tested in clinical trials in settings where they may inform specific interventions (e.g. surgery versus radiation for bladder or prostate cancer). Future studies should evaluate GA in specific tumor types and clinical settings with the ultimate goal to improve care delivery for older adults with GU cancers.

## Author contributions

All authors contributed to this study. All authors read and approved the final manuscript. Conceptualization: SS and AK. Literature review: SS, JM, and AK. Preparation of manuscript: SS, JM, and AK. All authors contributed to the article and approved the submitted version.
